# EFINUTRILES Study: Integrative Extra Virgin Olive Oil and Multimodal Lifestyle Interventions for Cardiovascular Health and SLE Management

**DOI:** 10.3390/nu17061076

**Published:** 2025-03-19

**Authors:** Rocío Gil-Gutiérrez, Irene Medina-Martínez, Miguel Quesada-Caballero, Francisco Javier de la Hera-Fernández, Mónica Zamora-Pasadas, Irene Cantarero-Villanueva, Luis Albendín-García, Vítor Parola, Blanca Rueda-Medina, María Correa-Rodríguez

**Affiliations:** 1 Department of Nursing, Faculty of Health Sciences, University of Granada, 18016 Granada, Spain; rogilgu@ugr.es (R.G.-G.); blarume@ugr.es (B.R.-M.); macoro@ugr.es (M.C.-R.); 2 CTS-436 Group, University of Granada, 18016 Granada, Spain; 3 Instituto de Investigación Biosanitaria (ibs.GRANADA), 18014 Granada, Spain; miguel.quesada.caballero.sspa@juntadeandalucia.es (M.Q.-C.); monikazamora@hotmail.com (M.Z.-P.); irenecantarero@ugr.es (I.C.-V.); 4 Sport and Health University Research Institute (iMUDS), University of Granada, 18007 Granada, Spain; 5 University of Granada, 18016 Granada, Spain; irene.medina.mrtnz@gmail.com; 6 Centro de Salud Albayda La Cruz, Calle Virgen de la Consolación, 12, Distrito Sanitario Granada-Metropolitano, Servicio Andaluz de Salud, 18015 Granada, Spain; 7 Systemic Autoimmune Diseases Unit, Hospital Universitario San Cecilio, 18016 Granada, Spain; 8 Systemic Autoimmune Diseases Unit, Hospital Universitario Virgen de las Nieves, 18014 Granada, Spain; 9 Department of Physical Therapy, University of Granada, 18016 Granada, Spain; 10 BIO-277 Group, University of Granada, 18016 Granada, Spain; 11 Casería de Montijo Health Center, Granda-Metropolitan Health District, Andalusian Health Service, Calle Virgen de la Consolación, 12, 18015 Granda, Spain; 12 Health Sciences Research Unit: Nursing (UICISA:E), Nursing School of Coimbra (ESEnfC), 3000 Coimbra, Portugal; vitorparola@esenfc.pt; 13 Portugal Centre for Evidence-Based Practice: A Joanna Briggs Institute Centre of Excellence (PCEBP), 3000 Coimbra, Portugal

**Keywords:** autoimmune diseases, cardiovascular risk, extra virgin olive oil, Mediterranean diet, physical exercise, systemic lupus erythematosus

## Abstract

Objectives: To analyze the effects of the combination of Extra Virgin Olive Oil (EVOO) supplementation and a health-related lifestyle intervention on disease activity and cardiovascular disease risk factors in Systemic Lupus Erythematosus (SLE). Methods: A total of 38 women with SLE were randomly assigned to EVOO (n = 9) and EVOO combined with multicomponent health promotion and physical exercise program (EVOO + HRLI) (n = 15) or control (CG) (n = 14) groups for 24 weeks. Baseline and post-intervention assessments were performed, collecting data on disease activity, accrual damage, blood biochemical parameters, arterial stiffness parameters, Framingham risk score, anthropometric and body composition measurements, and cardiovascular risk factors. Results: No changes in disease activity were observed in any group after the intervention. For cardiovascular risk, significant differences were observed in the intervention groups for systolic and mean blood pressure, with greater reductions in the EVOO + HRLI (*p* = 0.036 vs. *p* < 0.001; *p* = 0.017 vs. *p* < 0.001, respectively). The EVOO group showed significant reductions in BFM and BFP (*p* = 0.042, *p* = 0.022, respectively). The EVOO+ HRLI group also showed significant reductions in triglycerides (*p* < 0.001), Aix brachial (*p* = 0.037), central systolic blood pressure (*p* < 0.001), central pulse pressure (*p* = 0.05), body mass index (*p* = 0.006), body fat mass and skeletal muscle mass (*p* = 0.039) after the intervention. Conclusions: Our findings suggest that a multidisciplinary program integrating nutritional interventions, health education, and the promotion of regular physical activity in SLE patients has the potential to significantly improve cardiovascular risk factors and body composition parameters. Thus, integrating this approach into clinical practice alongside usual pharmacological treatments would be beneficial for SLE patients. Clinical Trial Registration: NCT05261529.

## 1. Introduction

Systemic Lupus Erythematosus (SLE) is an autoimmune condition with multisystemic involvement [1], characterized by the presence of autoantibodies targeting nuclear antigens, immune complex deposition, and a chronic inflammatory state [2]. It exhibits significant clinical heterogeneity [3], with manifestations ranging from mild to life-threatening, including hematological, cardiovascular, musculoskeletal, dermatological, renal, neuropsychiatric, and pulmonary symptoms, as well as photosensitivity and constitutional symptoms, all contributing to considerable morbidity and mortality.

Among this broad spectrum, cardiovascular diseases (CVDs) and their associated comorbidities constitute one of the main challenges. SLE patients face a two-to-tenfold higher risk of CVD compared to the general population [4]. Accelerated atherosclerosis, which leads to early CVD, occurs in up to 10% of SLE patients, making it six times more frequent than in the general population.

Beyond traditional factors for CVD (hypertension, diabetes, tobacco use, alcohol consumption, sedentary lifestyle), SLE-specific factors further exacerbate this risk, including elevated levels of inflammatory cytokines, circulating immune complexes, antiphospholipid antibodies, and dysregulation of T and B lymphocytes characteristic of SLE [5]. Consequently, SLE patients have a higher rate of endothelial dysfunction and impaired endothelial repair mechanisms [4]. Additionally, several unique features complicate the diagnosis and management of CVD in SLE, including its occurrence in young women, the absence of an inflammatory burden commonly associated with atherosclerosis, and a lack of protective response to statin treatment.

The specific mechanisms leading to the increased risk of CVD in SLE patients have not been fully characterized. However, it is clear that traditional CVD risk factors alone do not account for the higher risk observed in this population. It is highly probable that the immunological and inflammatory characteristics of SLE contribute significantly to this increased risk. As the understanding of SLE-specific factors contributing to CVD advances, there is a growing need for enhanced screening and monitoring of CVD risk, as well as the development of therapeutic strategies aimed at prevention.

In addition to the established pharmacological treatments currently available, dietary modifications have been shown to significantly improve CVD function in individuals with SLE [6]. Studies have found a lower prevalence of CVD events in SLE populations with high and sustained adherence to the Mediterranean Diet (MD) [7], which is characterized by the use of extra virgin olive oil (EVOO), its most representative component [8]. EVOO has well-documented anti-inflammatory, antioxidant, and antimicrobial properties. However, evidence in autoimmune disease cohorts is limited, and there is a lack of longitudinal studies establishing a causal relationship between MD, EVOO and the progression of SLE [9,10,11,12]. Similarly, although adapted moderate physical exercise (PE) interventions have been shown to be safe and feasible, offering several CVD health benefits [13], evidence remains limited due to variability in participants’ disease activity levels and the characteristics of the interventions implemented. Further studies are needed to explore how adherence to PE influences CVD risk and overall health in SLE. Moreover, interventions that incorporate health education and patient self-management, rather than isolated targeted approaches, may help sustain these benefits over time [14].

An integrative approach to managing health-related lifestyles [15,16] could improve clinical course for SLE patients, similar to the benefits reported in other non-communicable diseases such as diabetes or cancer. Although current evidence suggests the beneficial effects of both phenolic compounds in EVOO and adherence to PE on the progression of autoimmune conditions such as SLE [17,18], no intervention has combined these approaches.

The aim of this study was to analyze the effects of an intervention combining EVOO and a multicomponent health promotion and PE program on disease activity and CVD risk factors in SLE patients.

## 2. Materials and Methods

### 2.1. Study Design

We conducted a three-arm, prospective, randomized controlled clinical trial over twenty-four weeks, following the SPIRIT and CONSORT statements, as well as the Tidier checklist (Supplementary Table S1). This clinical trial has been registered at ClinicalTrials.gov (NCT05261529: Accessed on 1 March 2022).

### 2.2. Ethics and Consent

The study protocol was approved by the Biomedical Research Ethics Committee of Granada, Spain (2099-N-21) according to the Helsinki Declaration for biomedical research. All participants provided informed consent prior participation.

### 2.3. Study Design

The study population comprised SLE patients attending the outpatient clinic in the Systemic Autoimmune Diseases Unit at Hospital Universitario Clínico San Cecilio and Hospital Universitario Virgen de las Nieves (Granada, Spain), following the revised American College of Rheumatology (ACR), SLICC, or ACR/EULAR criteria from 2019. Inclusion criteria included (1) SLE diagnosis from at least one year; (2) stable SLEDAI-2K, with no treatment modifications, over the preceding three months; (3) sedentary or inactive individuals not participating in structured PE programs(defined as sitting for more than five hours per day, engaging in less than 300 min of weekly physical activity, or performing less than 60 min of structured PE per week); (4) medium to high adherence to the MD (7–14 points), measured using the 14-point MD adherence scale from the PREDIMED study [19]. Exclusion criteria comprised (1) end-stage SLE; (2) serum creatinine levels ≥ 1.5 mg/dL; (3) type 1 diabetes mellitus; (4) infection, trauma or surgery within the past six months; (5) SLICC rating of >5; (6) pregnancy or breastfeeding; (7) diagnosis of other autoimmune/inflammatory diseases; (8) participation in other guided PE programs; (9) contraindications for PE: psychiatric or cognitive disorders and acute or chronic conditions; (10) body mass index of (BMI) ≥ 40 kg/m^2^. After patients were screened through referrals (−t_1_), they were contacted and invited to the University of Granada where baseline assessment (t_0_), allocation (0) and consecutive face-to-face assessments (t_1_, t_2_), along with the PE intervention, were carried out.

As part of baseline assessment and before allocation, a nutritionist reminded participants of the basic principles of the MD through a one-hour non-face-to-face session to ensure adequate MD adherence during the intervention. Subsequently, patients were randomized using the Oxford Minimization and Randomization^®^ program (O’Callaghan, CA, USA), an open-source software designed for online minimization and randomization in clinical trials, allowing researchers to allocate participants in real time, ensuring balanced distribution of patient characteristics across study arms. Patients were allocated in a 1:1:1 ratio to intervention group 1 (EVOO group), intervention group 2 (EVOO + HRLI group), or control group (CG).

### 2.4. Intervention

Participants in the EVOO group added a daily supplement of 40 mL of EVOO, in a single dose, preferably at breakfast, to their habitual lifestyle (dietary and physical activity patterns), over twenty-four weeks. Participants allocated to the EVOO + HRLI group followed the same dietary intervention, and, after twelve weeks, they began a health-related lifestyle intervention (HRLI) which was combined with the dietary intervention for the remaining twelve weeks. Participants in the CG were encouraged to maintain their habitual lifestyle.

The EFINUTRILES program is an HRLI based on a multicomponent health promotion and PE program. More details of EFINUTRILES program components and specific EFINUTRILES PE program have been published in a previous protocol [20].

Baseline sociodemographic characteristics of the sample including age, tobacco use, alcohol consumption, MD adherence, and menopausal status were collected. Additionally, physical activity level was assessed using the International Physical Activity Questionnaire (IPAQ) [21]. Functional capacity was measured through the six-minute walking test (6 MWT), and handgrip muscle strength was evaluated using a TKK5101 Grip-D dynamometer (Takeya, Tokyo, Japan), following a standardized protocol [22].

### 2.5. Oucome Measures

#### 2.5.1. Main Outcome: SLEDAI2-K

Recognized as a reliable assessment tool for clinical trials and prognosis studies [23], SLEDAI2-K was calculated to assess SLE disease activity. This index integrates data from medical history, physical examination, organ-specific assessments and serological tests over the past month.

#### 2.5.2. Secondary Outcomes

##### Clinical Disease Parameters

Disease duration, pharmacological treatment (including immunosuppressants, glucocorticoids, and antimalarials), and comorbidities were recorded. Additionally, damage accrual score (SDI) was assessed using the Systemic Lupus International Collaborating Clinics/American College of Rheumatology SLICC/ACR Index, a validated tool developed by the ACR to measure cumulative SLE-related damage since disease onset, whether due to disease progression or its sequelae, across twelve organ systems [24]. Inflammatory and immunological markers’ (hsCRP, anti-double-stranded DNA antibodies anti-dsDNA, and complement factors C3 and C4) levels were also collected. The specific reagents and reference ranges used were as follows: hsCRP (CRP Test, Roche Diagnostics^®^, 0–5 mg/L), anti-dsdNA (Anti-dsDNA ELISA, Roche Diagnostics^®^, <30 IU/mL negative, 30–75 IU/mL equivocal, >75 IU/mL positive), C3 (C3 Complement Reagent, Siemens Healthcare^®^, 900–1800 mg/dL), and C4 (C4 Complement Reagent, Beckman Coulter^®^, 100–400 mg/dL). Samples were taken through blood tests after 8 h of fasting at 8:00.a.m by nursing professionals under standardized environmental conditions.

##### Cardiovascular Risk Factors

Under the same conditions described for clinical disease blood parameters, biochemical blood analyses were performed, including lipid profile and triglyceride levels (TG). Arterial stiffness was assessed using a portable arteriograph (TensioMed Arteriograph Tl2, Tensiomed Ltd., Budapest, Hungary). This non-invasive portable method has demonstrated adequate reliability and validity [25], and was used to measure the following parameters: pulse wave velocity (PWV), aortic augmentation index (AIx aortic), brachial augmentation index (AIx brachial), brachial pulse pressure (PP), central systolic blood pressure (SBPao), central pulse pressure (PPao), ankle–brachial index (ABI), brachial systolic blood pressure (SBP), brachial diastolic blood pressure (DBP) and mean blood pressure (MBP).

Anthropometric parameters including body mass index (BMI), waist and hip circumferences, and waist-to-hip ratio were measured using an inelastic measuring tape (Lufkin W606PM^®^, Parsippany, NJ, USA). Body composition parameters such as body fat mass (BFM), body fat percentage (BFP), skeletal muscle mass (SMM) and visceral fat area (VFA) were estimated using a bioelectrical impedance analyzer (Inbody 720, Biospace, Seoul, Republic of Korea). Additionally, the Framingham risk score, as a simplified, common, and applicable tool for predicting a person’s risk level of CVD over ten years [26,27,28] was calculated.

### 2.6. Data Collection

Participants were assessed at baseline (t_0_) prior to allocation (0), one week after three months of EVOO (just prior to EFINUTRILES program start for the EVOO + HRLI group) (t_1_), and after six months of intervention (t_2_) (Supplementary Table S2). All assessments were carried out by a single blinded researcher with five years of experience.

### 2.7. Data Analysis

All analyses were performed by a blinded researcher using IBM SPSS Statistics Version 25 (IBM Corp., Armonk, NY, USA). Results were considered statistically significant at *p* ≤ 0.05. Baseline sociodemographic and clinical data were described using preliminary descriptive statistics, with results presented as mean and standard deviation (m ± SD) for continuous data following a normal distribution, or median and interquartile range (median ± IQR) for continuous data with a non-normal distribution. Frequencies and percentages (n, %) were used for categorical data. The Kolmogorov–Smirnov test was used to assess the normality of the data. Baseline comparisons were conducted to evaluate group homogeneity using Student’s *t*-test or Mann–Whitney U test, as appropriate. Multivariate logistic regression analyses were carried out to assess the effects of the intervention on clinical disease characteristics and CVD risk factors. The magnitude of difference between groups was calculated using Cohen’s effect size values. Data analysis was based on the intention-to-treat principle, using imputation methods for any missing data.

## 3. Results

**Figure 1:**
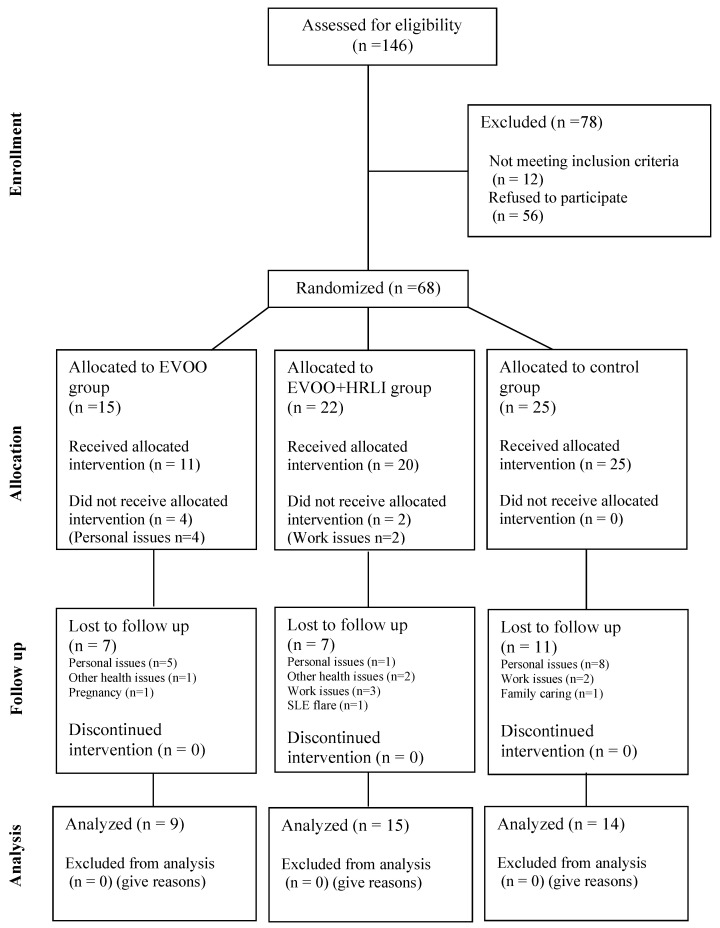
Participant flow diagram according to CONSORT guidelines (adapted). EVOO: extra virgin olive oil.

After randomization, 15, 22 and 25 participants were allocated to EVOO, EVOO + HRLI and CG. Considering dropouts, 9, 15 and 14 women ultimately completed their assigned intervention in EVOO, EVOO + HRLI and CG, respectively (Figure 1). The compliance rates for each group, considering the initial number of allocated participants and group dropouts, are as follows: a compliance rate of 53.33% for EVOO; 68.18% for the EVOO + HRLI group; and 56.00% for the CG. The mean compliance rate across all three groups was 59.68%.

Baseline characteristics are displayed in Table 1. The total sample comprised 38 women, median age 48.00 ± 16.50 years, with a median BMI of 23.10 ± 5.55 kg/m^2^, median disease duration of 14.00 ± 9.00 years, and a median met equivalents of task (METs) weekly consumption of 1234.00 ± 2341.00 METS/week. No significant differences were found between groups for any outcome of interest before intervention (*p* > 0.05 in all cases).

The effect of the intervention on disease activity and CVD risk factors in study groups are shown in Table 2 and Table 3 and Supplementary Table S3. ANOVA revealed no significant differences over time in the CG for any outcomes, except for brachial pulse wave velocity (PWV, *p* = 0.047).

Regarding clinical disease activity parameters, intra-group analysis showed no significant changes after intervention in disease activity (SLEDAI2-K), damage accrual, or inflammatory markers for any group (Supplementary Table S3).

In contrast, significant differences were observed in different CVD risk variables in the EVOO and EVOO + HRLI groups after intervention.

Specifically, significant changes were detected for SBP and MPB in both EVOO and EVOO + HRLI groups, with greater reductions in the EVOO + HRLI group (*p* = 0.036 vs. *p* < 0.001; *p* = 0.017 vs. *p* < 0.001, respectively). Significant differences were also found in TG (*p* < 0.001), AIx brachial (*p* = 0.037), SBPao (*p* < 0.001) and PPao (*p* = 0.005) for EVOO + HRLI group (Table 2). The greater change was found for SBPao for EVOO + HRLI group after intervention, with also the largest effect size observed (mean difference = 16.56, 95% CI 9.73 to 23.39, d = 1.059). Regarding anthropometric and body composition parameters, significant differences were found for BMI and SMM in the EVOO + HRLI group after intervention, with moderate effect sizes (d = 0.246, d = 0.330 and d = 0.275, respectively) (Table 2).

Between-group comparisons of changes at the end of the intervention for disease activity and CVD factors in SLE patients are also presented in Table 2 and Table 3 and Supplementary Table S3. The EVOO group exhibited a greater increase in BFM (mean difference 12.40; 95% CI 2.43, 22.37) and SMM (mean difference 0.90; 95% CI 0.51, 1.74) compared to the CG. Regarding changes between CG and EVOO + HRLI group, analysis also yielded a greater increase in BFP (mean difference 3.01; 95% CI 0.56, 5.98) and SMM (mean difference 1.15; 95% CI 0.26, 2.04). When comparing EVOO vs. EVOO + HRLI groups, statistically significant increases were found in SBPao (mean difference 12.72; 95% CI 4.40, 21.04) and BMI (mean difference 1.80; 95% CI 0.29, 3.31) (Table 2 and Table 3). No significant between-group differences were found for other disease activity parameters or CVD risk factors (all *p* > 0.05).

## 4. Discussion

In this study, we aimed to investigate for the first time the effects on an intervention based on EVOO nutritional supplementation alone or combined with a HRLI program including multicomponent health promotion, patient education and PE on disease activity and CVD risk factors in SLE patients.

The main findings suggest that the daily EVOO supplementation for twenty-four weeks leads to changes in arterial stiffness parameters (specifically, SBP and MBP) and body composition parameters (BFM and BFP) in SLE patients. Furthermore, when this supplementation was enhanced with an HRLI program, these changes in arterial stiffness were greater, and additional beneficial effects were observed including reduction in TG, arterial stiffness parameters (aix brachial, SBPao, PPao, SBP) and anthropometric and body composition outcomes (BMI, SMM). It should be noted that the only significant change observed for GC was an increase in PWV, which could suggest a worsening in the arterial stiffness increasing the CVD risk in this group of patients that did not modify their lifestyle.

None of the interventions conducted led to an improvement in SLE patients’ clinical parameters. Similarly to our findings, to date, no published study involving non-pharmacological, lifestyle-based interventions such as this one has reported reduction in SLE disease activity. Previous evidence [29,30,31] has shown that non-pharmacological approaches including interventions such as psychological therapies, exercise-based programs, electro-acupuncture or food supplementation, ranging from 5 to 52 weeks, can maintain or even improve health outcomes (e.g., fatigue, depression, pain, quality of life), but are ineffective in reducing SLE disease activity, assessed with tools such as the SLE Lupus Activity Index, Systemic Lupus Activity Measure, Systemic Lupus Erythematosus Disease Activity Index, SLEDAI-2K, Systemic Lupus Activity Measure-Revised or the Systemic Lupus Activity Questionnaire for Population Studies.

Regarding PE, although no intervention based solely on PE has either directly decreased SLE activity, solid evidence supports its beneficial effects on health outcomes [32], which correlates directly with disease activity progression [33]. Our results are in line with these findings, suggesting that while EVOO and HRLI including regular PE performance may not significantly reduce disease activity, they do not exacerbate it and improve health status at cardiovascular and anthropometric status, indicating a potential for safe inclusion in SLE management and enhancement strategies. The interventions conducted did not lead to changes in other clinical disease activity parameters investigated (SDI, hsCRP, anti-dsDNA, Complement C3 and C4). Previous studies have suggested that changes in inflammation biomarkers are likely to be modest at best in patients with autoimmune diseases who adopt PE, corroborating our findings [34].

Different changes were observed for cardiovascular risk variables depending on intervention received. In the EVOO group, statistically significant decreases in SBP and MBP were observed, with very large effect sizes. In the EVOO + HRLI group, in addition to these decreases (though a smaller size effect), statistically significant and clinically beneficial changes were also noted in TG, aix brachial, SBPao and PPao post-intervention, all with at least “large” effect sizes. These results align with previously published evidence supporting the beneficial effects on endothelial function and CVD risk factor outcomes derived from EVOO supplementation. An inverse association between EVOO consumption and intima-media thickness was described in individuals at high CVD risk [35,36], and an MD rich in EVOO improved endothelial function compared to a low-fat diet in prediabetes and diabetes [37], as well as SBP values in healthy populations [38]. In autoimmune conditions, preliminary in vitro and in vivo studies have suggested that EVOO and its polyphenols improve CVD symptoms in rheumatoid arthritis, inflammatory bowel disease, multiple sclerosis, SLE and psoriasis [39], though further investigation is needed as most results are from cross-sectional studies. This study contributes new results addressing the effects of unenriched or modified EVOO supplementation on cardiovascular health on SLE through a longitudinal design, showing beneficial results in key parameters for CVD management, the leading actual cause of mortality in this pathology [40]. Superior benefits observed in EVOO + HRLI group reinforce the well-known but not fully elucidated role of PE as a protective agent against CVD risk in autoimmune conditions [41,42].

Anthropometrics and body composition parameters require a detailed explanation. Given the increased calorie intake due to the EVOO supplementation, a gain in body fat content could be expected, as potentially reflected in BFM and BFP increases in EVOO + HRLI group. However, these results have not achieved statistically significant values, requiring further observation and validation. Conversely, attention should be given to the statistically significant decreases reported in BFM and BFP for the EVOO group, consistent with previous studies [43]. All these parameters have a direct and proven effect on reducing CVD risk, emphasizing the importance of dietary fat quality. The MD, characterized by limited consumption of meat, dairy products, and a low intake of saturated fat (about 8% of total caloric value, TCV) favors a relatively high intake of total fat (25–35% TCV) primarily from EVOO, nuts, seeds, and whole grain germs [44]. This dietary pattern is central to the widely documented benefits in both healthy and autoimmune disease cohorts [45].

Secondly, in the EVOO + HRLI group, the intervention might initially appear to have exerted a negative effect, as reflected in a significant increase in BMI. Despite BMI being the most widely used indicator for estimating body mass, its correlation with body fat is relatively poor, since it does not distinguish between muscle and fat mass [46,47,48]. Despite the observed significant increase in BMI from baseline, this increase is due to an HRLI favoring a body composition remodulation, yielding a significant increase in SMM. These increases in both SMM and BMI can explain the slight increase in fat composition observed in the EVOO + HRLI group, as gaining muscle often requires a caloric surplus, which can also lead to a normal and temporal fat gain. Changes in body composition focused on SMM increases have been associated with greater survival, reduced disease activity and decreased comorbidities, including CVD, in SLE patients [49].

Thus, the results of this study could have importance at the clinical level since they support the potential of a non-pharmacological and non-invasive intervention based on natural supplementation with EVOO that improves different parameters of cardiovascular risk and enhances anthropometrics and body composition parameters in SLE patients. Additionally, the study highlights the need for multidisciplinary programs that integrate nutritional interventions, health education, and the promotion of regular physical activity in SLE patients, as results in the combined HRLI and EVOO group are greater than for the EVOO group. This randomized controlled trial is the first to develop a combined intervention including EVOO supplementation and a multicomponent physical activity and health promotion program in SLE patients, providing valuable knowledge improving health system sustainability and quality of care provided by health professionals.

This study has some potential limitations. Due to the small sample size and the well-characterized and stable cohort of female SLE patients, statistical power is limited, and results may not be generalizable, requiring further validation in larger study cohorts including male patients. It is important to note the unique challenges we faced in accessing the population with SLE during the post-pandemic period, which significantly limited recruitment across all studies. In addition, the compliance rates observed in this study, with a mean of 59.68%, are relatively low. This can be attributed to sudden changes in the course of the disease that occur in SLE patients who often experience disease flares or exacerbation of symptoms, needing specialized medical attention on many occasions. These factors can greatly impact their ability to manage daily life and therefore adhere to study protocols. Despite these limitations, the preliminary results provide valuable insights and establish a starting point to further validation.

In contrast, this study has various key strengths. The first is the guarantee of the stability of the EVOO composition used through the intervention period, verified through various laboratory analyses conducted by the supplier DCOOP (S. Coop. And), which confirmed the absence of changes in the components of the supplied EVOO. In addition, reminders such as pictograms, drinking cups, and group messages on messaging platforms were employed weekly to ensure adherence. Finally, the inclusion of a control group, in which no changes were reported, reinforces the results. Although one important limitation is the inability to blind patients in the intervention groups, this was counteracted by a randomization and allocation process conducted by blinded researchers and the use of valid, reliable, objective instruments, which should eliminate any risk of response bias.

## 5. Conclusions

Our results suggest that EVOO nutritional supplementation induces beneficial changes in cardiovascular risk parameters in SLE patients. When this supplementation is enhanced by an HRLI including a PE program and health education, the observed changes are even greater, adding beneficial changes in the triglyceride levels, other arterial stiffness parameters (aix brachial, SBPao, PPao, SBP, MBP), and anthropometric and body composition measures (BMI, SMM). This non-invasive, multidisciplinary approach, complementary to current drug treatments, would provide promising results for managing the clinical course and comorbidities of SLE, in which CVD risk is a primary concern. Larger cohorts are necessary to confirm these preliminary findings.

## Figures and Tables

**Table 1:** Sociodemographic characteristics, clinical data, pharmacologic treatment, comorbidities, and anthropometry and physical activity data of participants.

	Total (n = 38)	EVOO Group (n = 9)	EVOO + HRLI Group (n = 15)	Control Group (n = 14)	*p* Value (Control vs. EVOO)	*p* Value (Control vs. EVOO + HRLI)	*p* Value (EVOO vs. EVOO + HRLI)
Characteristics							
Age (years) ^A^	48.00 ± 16.50	48.00 ± 12.50	48.00 ± 32.00	47.00 ± 16.25	0.258	0.670	0.295
Current tobacco use (n) ^B^	13 (34.20)	3 (33.30)	3 (20.00)	7 (50.00)	0.235 ^C^		
Alcohol consumption (n) ^B^	19 (50.00)	4 (44.40)	8 (53.30)	7 (50.00)	0.915 ^C^		
MedDiet score (points) ^A^	8.00 ± 2.00	9.00 ± 3.00	9.00 ± 2.25	8 ± 6.00	0.676	0.288	0.704
Menopause (n) ^B^	19 (50.00)	4 (44.40)	9 (60.00)	6 (42.90)	0.607 ^C^		
Clinical data ^A^							
Disease duration (years)	14.00 ± 9.00	18.00 ± 8.00	13.50 ± 13.25	10.00 ± 10.50	0.768	0.887	0.914
SLEDAI-2K (points)	4.00 ± 6.00	4.00 ± 7.50	5.50 ± 8.00	3.00 ± 7.00	0.078	0.127	0.349
SDI (points)	1.00 ± 1.50	0.00 ± 1.50	1.00 ± 2.25	1.00 ± 1.25	0.205	0.083	0.907
Treatment (n) ^B^							
Antimalarial use	31 (83.8)	9 (100.0)	13 (92.9)	9 (64.3)	0.039 ^C^		
Immunosuppressor use	14 (37.8)	2 (22.2)	4 (28.6)	8 (57.1)	0.160 ^C^		
Corticoid use	15 (40.5)	1 (11.1)	7 (50.0)	7 (50.0)	0.118 ^C^		
Comorbidities (n) ^B^							
Hypertension	11 (29.7)	0 (0)	7 (50.0)	4 (28.6)	0.080 ^C^		
Diabetes	2 (5.4)	1 (11.1)	0 (0)	1 (7.1)	0.483 ^C^		
Dyslipidaemia	5 (13.5)	3 (33.3)	2 (14.3)	0 (0)	0.074 ^C^		
Anthropometry ^A^							
Weight (kg)	59.30 ± 16.08	60.50 ± 30.60	59.10 ± 15.30	58.20 ± 18.95	0.308	0.612	0.092
Height (m)	1.50 ± 0.09	1.59 ± 0.10	1.56 ± 0.08	1.61 ± 0.09	0.575	0.158	0.343
BMI (kg/m^2^)	23.10 ± 5.55	24.20 ± 12.45	23.10 ± 4.35	22.40 ± 4.50	0.178	0.613	0.145
Physical activity ^A^							
(IPAQ score; Mets/week)	1234.00 ± 2341.00	792.00 ± 3139.50	1350.000 ± 2520.00	1053.00 ± 1970.50	0.605	0.616	0.336
Sedentary (min/day)	435.00 ± 360.00	450.00 ± 450.00	439.00 ± 300.00	420.00 ± 390.00	0.734	0.648	0.983
Handgrip (kg)	256.76 ± 9.65	27.46 ± 9.52	24.43 ± 5.11	31.13 ± 7.94	0.318	0.115	0.649
Functional capacity (6 MWT; meters)	570.00 ± 118.25	534.00 ± 107.50	579.00 ± 97.00	582.50 ± 133.50	0.326	0.709	0.487

Data are expressed as ^A^ median and interquartile range (median ± IQR) for continuous data or as ^B^ frequency (%) for qualitative variables. ^C^ Student’s *t*-tests (continuous variables) or chi squared tests (categorical variables). Abbreviations: 6 MWT = Six-Minute Walking Test; BMI = Body Mass Index; IPAQ = International Physical Activity Questionnaire; SDI = SLICC/ACR Damage Index; SLEDAI-2K = Systemic Lupus Erythematosus Disease Activity Index 2000; EVOO = Extra Virgin Olive Oil; HRLI = Health-Related Lifestyle Intervention.

**Table 2:** Biochemical measures and arterial stiffness parameters as cardiovascular risk factors at baseline and changes after 24 weeks by groups.

						Inter-Group Difference in Change		
	Baseline	24-Weeks	*p* Value	Cohen’s d Within-Group	Within-Group Change	Control vs. EVOO	Control vs. EVOO + HRLI	EVOO vs. EVOO + HRLI
Biochemical parameters								
TC (mg/dL)								
EVOO	183.00 ± 46.5	203.00 ± 52.25	0.22	0.77	−23.50 (−59.96, 9.96)	9.11 (−26.8, 45.12)	−15.30 (−36.08, 5.46)	24.42 (−51.34, 2.49)
EVOO + HRLI	202.00 ± 18.25	193.00 ± 48.50	0.86	0.03	0.92 (−10.58, 12.42)			
Control	1184.00 ± 33.7	196.00 ± 28.00	0.11	0.48	−14.38 (−33.05, 4.28)			
TG (mg/dL)								
EVOO	74.00 ± 36.00	73.500 ± 95.50	0.221	0.439	−36.87 (−101.80, 28.05)	23.15 (−27.79, 74.09)	−26.97 (−45.28, −8.66)	−50.12 (−97.09, −3.15)
EVOO + HRLI	67.00 ± 36.00	60.00 ± 43.50	<0.001	0.539	13.25 (7.30, 19.19)			
Control	60.00 ± 36.00	72.00 ± 57.50	0.117	0.317	−13.72 (−31.47, 4.02)			
HDL-C (mg/dL)								
EVOO	63.00 ± 15.0	61.500 ± 23.25	0.961	0.008	−0.12 (−5.97, 5.72)	−2.37 (−9.54, 4.79)	−1.10 (−8.44, 6.24)	1.27 (−6.38, 8.93)
EVOO + HRLI	70.00 ± 13.50	65.00 ± 24.00	0.606	0.15	−1.40 (−7.34, 4,54)			
Control	55.00 ± 20.00	60.00 ± 11.00	0.304	0.27	−2.50 (−7.60, 2.60)			
LDL-C (mg/dL)								
EVOO	106.50 ± 35.25	122.50 ± 42.5	0.145	0.901	−22.25 (−54.30, 9.80)	15.916 (−12.757, 44.590)	−2.20 (−22.92, 18.50)	−18.12 (−50.65, 14.40)
EVOO + HRLI	115.00 ± 33.50	121.00 ± 43.75	0.564	0.195	−4.12 (−20.21, 11.96)			
Control	114.50 ± 32.75	113.50 ± 23.25	0.271	0.360	−10.33 (−29.94, 9.28)			
Arterial stiffness parameters								
AIx aortic (%)								
EVOO	40.40 ± 8.95	32.85 ± 17.28	0.464	0.385	−24.12 (−97.72, 49.47)	20.55 (−28.77, 69.88)	−0.49 (−10.17, 9.18)	−21.04 (−69.77, 24.68)
EVOO + HRLI	23.80 ± 28.60	24.80 ± 21.05	0.517	0.195	−3.07 (−8.86, 2.70)			
Control	33.46 ± 29.57	35.55 ± 18.12	0.369	0.254	−3.57 (−11.86, 4.72)			
PWV (m·s^−1^)								
EVOO	8.80 ± 1.12	9.10 ± 1.40	0.125	0.105	−0.21 (−2.64, 2.22)	−1.01 (−3.24, 1.21)	−0.96 (−2.37, 0.44)	0.05 (−1.88, 1.99)
EVOO + HRLI	8.60 ± 3.00	7.60 ± 2.97	0.517	0.091	−0.26 (−1.12, 0.59)			
Control	7.40 ± 1.53	7.85 ± 2.55	0.047	0.714	−1.22 (−2.4, −0.017)			
AIx brachial (%)								
EVOO	−24.65 ± 57.58	−25.40 ± −41.63	0.824	0.043	−1.12 (−11.82, 9.58)	−18.83 (−55.27, 17.60)	−34.68 (−71.51, 2.14)	15.84 (−0.16, 31.86)
EVOO + HRLI	5.50 ± 17.63	−11.20 ± 11.42	0.037	0.929	14.72 (1.18, 28.26)			
Control	−7.400 ± 57.03	−4.15 ± 40.70	0.245	0.198	−19.95 (−55.38, 15.46)			
SBPao (mmHg)								
EVOO	114.11 ± 34.85	114.45 ± 25.00	0.178	0.223	3.83 (−1.98, 9.65)	−0.89 (−9.29, 7.51)	−13.61 (−22.45, −4.77)	12.72 (4.40, 21.04)
EVOO + HRLI	130.95 ± 33.45	104.75 ± 21.40	<0.001	1.059	16.56 (9.73, 23.39)			
Control	117.20 ± 23.45	113.35 ± 9.70	0.356	0.206	2.94 (−3.70, 9.59)			
PPao (mmHg)								
EVOO	48.50 ± 15.55	45.75 ± 10.85	0.321	0.390	27.90 (−30.53, 86.34)	−26.67 (−85.25, 31.90)	−7.07 (−13.71, −0.44)	−19.59 (−78.13, 38.94)
EVOO + HRLI	45.10± 15.20	43.35 ± 15.95	0.005	0.970	8.31 (3.41, 13.21)			
Control	42.75 ± 15.43	44.60 ± 10.55	0.616	0.147	1.23 (−3.96, 6.43)			
ABI								
EVOO	1.16 ± 0.09	1.21 ± 0.12	0.351	0.502	−3.76 (−12.65, 5.13)	3.04 (−5.99, 12.07)	−0.65 (−3.13, 1.82)	−3.70 (−12.59, 5.19)
EVOO + HRLI	1.18 ± 0.17	1.23 ± 0.14	0.296	0.485	−0.05 (−0.17, 0.05)			
Control	1.15 ± 0.13	1.21 ± 0.08	0.576	0.230	1.23 (−3.96, 6.43)			
SBP (mmHg)								
EVOO	130.50 ± 26.25	113.00 ± 23.50	0.036	1.029	15.87 (1.35, 30.39)	−9.94 (−25.03, 5.13)	2.73 (−9.66, 4.18)	7.20 (−7.44, 21.85)
EVOO + HRLI	112.00 ± 13.00	109.00 ± 21.00	<0.001	0.655	8.66 (5.14, 12.19)			
Control	120.50 ± 11.75	111.00 ± 10.50	0.061	0.611	5.92 (−0.32, 12.17)			
DBP (mmHg)								
EVOO	70.00 ± 19.50	70.50 ± 17.25	0.123	0.825	7.25 (−2.53, 17.03)	−5.53 (−15.63, 4.56)	−1.75 (−7.30, 3.79)	3.78 (−6.42, 13.98)
EVOO + HRLI	66.00 ± 12.0	66.00 ± 15.00	0.108	0.365	3.46 (−0.86, 7.79)			
Control	71.00 ± 11.00	68.50 ± 8.50	0.358	0.278	1.71 (−2.17, 5.60)			
MBP (mm/Hg)								
EVOO	91.50 ± 20.50	84.50 ± 19.50	0.017	0.884	9.37 (2.29, 16.45)	−6.44 (−14.07, 1.18)	−3.67 (−8.23, 0.89)	2.77 (−4.49, 10.04)
EVOO + HRLI	80.00 ± 13.00	82.50 ± 13.50	<0.001	0.612	6.60 (3.97, 9.22)			
Control	89.00 ± 11.00	82.50 ± 5.50	0.135	0.142	2.92 (−1.03, 6.89)			
Framingham risk score								
EVOO	3.60 ± 7.90	3.90 ± 7.87	0.934	0.018	−0.08 (−2.55, 2.39)	0.51 (−1.97, 3.01)	0.95 (−0.30, 2.21)	0.43 (−2.04, 2.91)
EVOO + HRLI	3.15 ± 3.38	3.15 ± 4.10	0.223	0.172	−0.520 (−1.41, 0.37)			
Control	2.80 ± 5.23	1.60 ± 5.30	0.363	0.098	0.43 (−0.57, 1.43)			

Abbreviations: ABI = ankle–brachial index; AIx aortic = aortic augmentation index; AIx brachial = brachial augmentation index; DBP = brachial diastolic blood pressure; HDL-C = high-density lipoprotein; LDL-C = low-density lipoprotein; MBP = mean blood pressure; PPao = pulmonary artery occlusion pressure; PWV = pulse wave velocity; SBP = brachial systolic blood pressure; SBPao = central systolic blood pressure; TC = total cholesterol; TG = triglyceride; EVOO = Extra Virgin Olive oil; HRLI = Health-Related Lifestyle Intervention.

**Table 3:** Anthropometrics and body composition parameters as cardiovascular risk factors at baseline and changes after 24 weeks by groups.

							Inter-Group Difference in Change	
	Baseline	24-Weeks	*p* Value	Cohen’s d Within-Group	Within-Group Change	Control vs. EVOO	Control vs. EVOO + HRLI	EVOO vs. EVOO + HRLI
Anthropometric parameters								
BMI (kg/m^2^)								
EVOO	24.20 ± 12.45	25.20 ± 10.75	0.31	0.140	0.87 (−1.01, 2.76)	−1.43 (−3.37, 0.50)	0.36 (−0.48, 1.21)	1.80 (0.29, 3.31)
EVOO + HRLI	23.00 ± 4.35	23.40 ± 3.60	0.006	0.330	−0.92 (−153, −0.30)			
Control	21.75 ± 4.50	22.90 ± 5.63	0.085	0.164	−0.55 (−1.20, 0.08)			
Waist circumference (cm)								
EVOO	78.00 ± 28.50	77.50 ± 22.75	0.746	0.049	0.77 (−4.57, 6.12)	0.54 (−5.95, 7.04)	0.08 (−5.01, 5.18)	−0.45 (5.64, 4.73)
EVOO + HRLI	73.00 ± 15.00	75.00 ± 13.50	0.380	0.129	1.23 (−1.68, 4.14)			
Control	77.75 ± 14.00	78.50 ± 15.88	0.531	0.137	1.32 (−3.11, 5.76)			
Hip circumference (cm)								
EVOO	100.00 ± 33.50	105.00 ± 25.25	0.979	0.003	−0.05 (−4.73, 4.62)	2.05 (−4.05, 8.16)	7.95 (−5.35, 21.25)	5.89 (−10.50, 22.30)
EVOO + HRLI	93.50 ± 11.00	97.00 ± 12.00	0.334	0.292	−5.95 (−18.72, 6.81)			
Control	96.00 ± 15.50	93.75 ± 17.88	0.361	0.221	2.00 (−2.56, 6.56)			
Body composition parameters								
Body fat mass (kg)								
EVOO	19.80 ± 23.00	20.10 ± 20.05	0.042	0.246	2.82 (0.12, 5.52)	4.45 (−3.33, 12.24)	12.40 (2.43, 22.37)	−7.94 (−16.21, 0.31)
EVOO + HRLI	17.80 ± 9.40	15.40 ± 9.10	0.603	0.085	−0.46 (−2.31, 1.39)			
Control	14.35 ± 9.75	16.85 ± 12.73	0.251	0.121	−0.89 (−2.49, 0.71)			
Body fat (%)								
EVOO	32.80 ± 18.90	32.40 ± 17.20	0.022	0.200	2.90 (0.53, 5.2)	−0.19 (−5.92, 5.54)	3.01 (0.56, 5.98)	3.21 (−2.60, 9.02)
EVOO + HRLI	28.90 ± 8.00	29.90 ± 10.10	0.115	0.192	−1.21 (−3.53, 1.11)			
Control	25.00 ± 9.07	26.70 ± 12.25	0.082	0.194	−1.65 (−3.55, 0.24)			
Skeletal muscle mass (kg)								
EVOO	23.60 ± 4.55	24.00 ± 6.25	0.078	0.185	−0.60 (−1.27, 0.07)	0.90 (0.51, 1.74)	1.15 (0.26, 2.04)	−0.25 (−1.26, 0.75)
EVOO + HRLI	20.30 ± 5.00	21.20 ± 4.50	0.039	0.275	−0.85 (−1.65, −0.05)			
Control	23.55 ± 6.28	22.80 ± 5.50	0.273	0.084	0.30 (−0.26, 0.86)			
Visceral fat area (cm^2^)								
EVOO	96.50 ± 98.55	99.90 ± 86.10	0.516	0.080	3.92 (−9.38, 17.22)	−4.27 (−19.04, 10.48)	1.82 (−10.44, 14.09)	6.10 (−8.31, 20.52)
EVOO + HRLI	69.20 ± 27.70	69.30 ± 26.20	0.606	0.084	−2.18 (−11.04, 6.68)			
Control	72.95 ± 44.38	82.15 ± 37.40	0.935	0.010	−0.35 (−9.70, 8.98)			

Abbreviations: BMI = body mass index; EVOO = Extra Virgin Olive Oil; HRLI = Health-Related Lifestyle Intervention.

## Data Availability

Data supporting the findings obtained are available on appropriate request from the corresponding author, not being publicly available for ethical reasons.

## Supplementary Materials

The following supporting information can be downloaded at: https://www.mdpi.com/article/10.3390/nu17061076/s1, Table S1: The TIDieR (Template for Intervention Description and Replication) Checklist; Table S2: Study intervention; Table S3: Clinical disease activity parameters at baseline and changes after 24 weeks by groups.

## Abbreviations

The following abbreviations are used in this manuscript:

6 MWT	Six-minute walking test
ABI	Ankle–brachial index
ACR	American College of Rheumatology
Anti-dsDNA	Anti-double stranded DNA antibodies
BFM	Body fat mass
BFP	Body fat percentage
BMI	Body mass index
CG	Control group
CVD	Cardiovascular disease
DBP	Brachial diastolic blood pressure
EVOO	Extra virgin olive oil
HDL-C	High-density lipoprotein
HRLI	Health-Related Lifestyle Intervention
hsCRP	High-sensitivity C-reactive protein
IQR	Interquartile range
IPAQ	International Physical Activity Questionnaire
LDL-C	Low-density lipoprotein
MBP	Median blood pressure
MD	Mediterranean Diet
PP	Brachial pulse pressure
PPao	Central pulse pressure
PE	Physical exercise
PWV	Pulse wave velocity
SBP	Brachial systolic blood pressure
SBPao	Central systolic blood pressure
SDI	SLICC/ACR damage accrual score index
SLE	Systemic Lupus Erythematosus
SLEDAI2-K	Systemic Lupus Erythematosus Disease Activity Index 2000
SLICC	Systemic Lupus International Collaborating Clinics
SMM	Skeletal muscle mass
TC	Total cholesterol
TG	Triglyceride
VFA	Visceral fat area
