# Supplementary material for: EFINUTRILES Study: Integrative Extra Virgin Olive Oil and Multimodal Lifestyle Interventions for Cardiovascular Health and SLE Management
Source: Nutrients. 2025 Mar 19;17(6):1076. doi: 10.3390/nu17061076 (PMC11944431; doi:10.3390/nu17061076)
Supplement: Supplementary file 1 [file nutrients-17-01076-s001.zip › Table S2.pdf]

	STUDY INTERVENTION				
	**Enrollment**	**Baseline**	**Allocation**	**During intervention**	**End of intervention**
**TIMEPOINT**	**$-t_1$**	**t_0**	**0**	**t_1**	**t_2**
**ENROLMENT:**					
Allocation			X		
Elegibility	X				
Screenning	X				
Informed consent	X				
MD reinforcement		X			
**INTERVENTIONS**					
Control group					
Intervention group 1: EVOO				X	
Intervention group 2: EVOO+HRLI				X	
**ASSESSMENTS**					
Sociodemographic characteristics		X			
Disease duration		X			
Pharmacological prescription		X			
Comorbidities		X			
Physical activity level		X			
Functional capacity		X			
Handgrip strentgh		X			
SLE disease activity		X			X
SLE damage		X			X
Disease activity blood parameters		X			X
Biochemical blood parameters		X			X
Arterial Stifness		X			X
Framingham Score		X			X
Anthropometry		X			X
Body composition		X			X
