# Supplementary material for: EFINUTRILES Study: Integrative Extra Virgin Olive Oil and Multimodal Lifestyle Interventions for Cardiovascular Health and SLE Management
Source: Nutrients. 2025 Mar 19;17(6):1076. doi: 10.3390/nu17061076 (PMC11944431; doi:10.3390/nu17061076)
Supplement: Supplementary file 1 [file nutrients-17-01076-s001.zip › Table S3.pdf]

Table S3. Clinical disease activity parameters at baseline and changes after 24 weeks by groups.

	Baseline	24-weeks	P value	Cohen's d Within-Group	Within-Group Change	Intergroup Difference in Change		
						Control vs EVOO	Control vs EVOO+HRLI	EVOO vs EVOO+HRLI
**SLEDAI-2K (points)**								
EVOO	4.00±7.50	8.00±11.0	0.500	0.000	0.00 (-2.20, 2.20)	-1.69 (-5.04, 1.66)	0.61 (-3.41, 4.64)	2.30 (-1.98, 6.60)
EVOO+HRLI	4.00±6.00	6.00±11.0	0.084	0.346	-2.30 (-5.72, 1.11)			
Control	3.00±7.00	3.00±8.00	0.085	0.325	-1.69 (-4.21, 0.82)			
**SDI (points)**								
EVOO	0.00±1.50	0.00±0.50	0.211	0.645	0.77 (-0.54, 2.09)	-0.42 (-1.82, 0.98)	0.04 (-0.90, 1.00)	0.47 (-0.96, 1.90)
EVOO+HRLI	1.00±2.20	1.00±2.00	0.392	0.243	0.30 (-0.44, 1.06)			
Control	1.00±1.25	0.00±1.00	0.266	0.496	0.35 (-0.30, 1.02)			
**hsCRP (mg/dL)**								
EVOO	2.11±2.70	2.32±3.35	0.663	0.131	-0.21 (-1.28, 0.86)	2.49 (-0.00, 4.99)	2.24 (-0.37, 4.87	-0.24 (-1.67, 1.18)
EVOO+HRLI	1.30±0.50	1.26±0.80	0.943	0.024	0.03 (-1.06, 1.13)			
Control	4.06±7.00	1.77±1.75	0.057	0.701	2.28 (-0.07, 4.64)			
**Anti-dsDNA (IU/mL)**								
EVOO	22.0±53.38	31.50±50.00	0.125	0.059	3.08 (-1.22 7.39)	-30.92 (-108.77, 46.93)	-41,37 (-101.76, 19.02)	-10.45 (-41.24, 20,34)
EVOO+HRLI	3.00±30.50	9.92 ±14.70	0.225	0.471	13.53 (-9.80 36.87)			
Control	8.45±9.93	41.32±2.69	0.393	0.451	-27.83 (-100.20,44.52)			
**Complement C3 (mg/dL)**								
EVOO	119.70±43.2	101.50±35.05	0.588	0.149	4.47 (-13.83, 22.78)	-6.48 (-26.05, 13.07)	-4.35 (-17.32, 8.62)	2.13 (-17.62, 21.89)
EVOO+HRLI	88.95±24.13	79.00±27.1	0.618	0.107	2.34 (-7.62, 12.30)			
Control	89.85±47.70	79.85±33.07	0.649	0.071	-2.00 (-11.44, 7.43)			

**Complement C4 (mg/dL)**								
EVOO	26.00±16.15	19.50±14.95	0.179	0.206	2.07 (-1.17, 5.32)	-1.25 (-5.28, 2.77)	-0.95 (-4.54, 2.64)	0.30 (-3.53, 4.13)
EVOO+HRLI	17.10±12.00	16.40±10.40	0.148	0.252	1.77 (-0.72, 4.27)			
Control	19.80±13.02	19.55±11.40	0.539	0.097	0.82 (-2.03, 3.68)			

Data are expressed as median and interquartile range (median ± IQR) for continuous data Abbreviations: Anti-dsDNA= Anti-double stranded DNA antibodies; hsCRP= high-sensitivity C-reactive protein; SDI= SLICC/ACR Damage Index; SLEDAI-2K= Systemic Lupus Erythematosus Disease Activity Index 2000.
